# Peri‐ictal hypoxia is related to extent of regional brain volume loss accompanying generalized tonic‐clonic seizures

**DOI:** 10.1111/epi.16615

**Published:** 2020-07-19

**Authors:** Luke A. Allen, Ronald M. Harper, Sjoerd B. Vos, Catherine A. Scott, Nuria Lacuey, Laura Vilella, Joel S. Winston, Benjamin P. Whatley, Rajesh Kumar, Jennifer Ogren, Jaison S. Hampson, Sandhya Rani, Gavin P. Winston, Louis Lemieux, Samden D. Lhatoo, Beate Diehl

**Affiliations:** ^1^ Department of Clinical and Experimental Epilepsy UCL Institute of Neurology University College London London UK; ^2^ Epilepsy Society MRI Unit Chalfont St Peter UK; ^3^ The Center for SUDEP Research National Institute of Neurological Disorders and Stroke Bethesda MD USA; ^4^ UCLA Brain Research Institute Los Angeles CA USA; ^5^ Department of Neurobiology David Geffen School of Medicine at UCLA Los Angeles CA USA; ^6^ Centre for Medical Image Computing University College London London UK; ^7^ Neuroradiological Academic Unit UCL Institute of Neurology University College London London UK; ^8^ Department of Clinical Neurophysiology National Hospital for Neurology and Neurosurgery UCLH London UK; ^9^ Department of Neurology University of Texas Health Sciences Center at Houston Houston TX USA; ^10^ Department of Anaesthesiology David Geffen School of Medicine at UCLA Los Angeles CA USA; ^11^ Division of Neurology Department of Medicine Queen's University Kingston Ontario Canada

**Keywords:** epilepsy, hypoxia, MRI, SUDEP

## Abstract

**Objectives:**

Hypoxia, or abnormally low blood‐oxygen levels, often accompanies seizures and may elicit brain structural changes in people with epilepsy which contribute to central processes underlying sudden unexpected death in epilepsy (SUDEP). The extent to which hypoxia may be related to brain structural alterations in this patient group remains unexplored.

**Methods:**

We analyzed high‐resolution T1‐weighted magnetic resonance imaging (MRI) to determine brain morphometric and volumetric alterations in people with generalized tonic‐clonic seizures (GTCS) recorded during long‐term video‐electroencephalography (VEEG), recruited from two sites (n = 22), together with data from age‐ and sex‐matched healthy controls (n = 43). Subjects were sub‐divided into those with mild/moderate (GTCS‐hypox‐mild/moderate, n = 12) and severe (GTCS‐hypox‐severe, n = 10) hypoxia, measured by peripheral oxygen saturation (SpO_2_) during VEEG. Whole‐brain voxel‐based morphometry (VBM) and regional volumetry were used to assess group comparisons and correlations between brain structural measurements as well as the duration and extent of hypoxia during GTCS.

**Results:**

Morphometric and volumetric alterations appeared in association with peri‐GTCS hypoxia, including volume loss in the periaqueductal gray (PAG), thalamus, hypothalamus, vermis, cerebellum, parabrachial pons, and medulla. Thalamic and PAG volume was significantly reduced in GTCS patients with severe hypoxia compared with GTCS patients with mild/moderate hypoxia. Brainstem volume loss appeared in both hypoxia groups, although it was more extensive in those with severe hypoxia. Significant negative partial correlations emerged between thalamic and hippocampal volume and extent of hypoxia, whereas vermis and accumbens volumes declined with increasing hypoxia duration.

**Significance:**

Brain structural alterations in patients with GTCS are related to the extent of hypoxia in brain sites that serve vital functions. Although the changes are associative only, they provide evidence of injury to regulatory brain sites related to respiratory manifestations of seizures.

**Key points**

- Seizures, especially generalized tonic‐clonic (GTC), are commonly accompanied by hypoxia
- It is unknown whether seizure‐related hypoxia is related to brain structural alterations seen in people who experience GTC seizures (GTCS)
- Thalamic, periaqueductal gray (PAG), hippocampal, and brainstem volume loss in GTCS patients is related to the extent of peri‐ictal hypoxia
- The current findings provide evidence of injury to regulatory brain sites related to respiratory manifestations of seizures

## 1 BACKGROUND

Patients with epilepsy who succumb to sudden expected death in epilepsy (SUDEP), and those who are at elevated risk, show extensive regional brain structural changes, especially in gray matter areas responsible for autonomic, respiratory, and sensory regulation.^1^, ^2^ The processes inducing these alterations, as well as the temporal course of injury and precise relationships with SUDEP, remain poorly understood.

Prospective imaging studies investigating links between SUDEP risk factors observed in epilepsy monitoring units (such as physiologic, autonomic, and respiratory alterations) and brain structural changes are lacking. Demonstration of such links may shed light on mechanisms underlying the fatal scenario, reveal markers for early detection of SUDEP risk, and guide the tailoring of appropriate interventions to prevent catastrophic events.

The processes leading to SUDEP appear to involve centrally mediated postictal patterns of respiratory and cardiovascular dysfunction; namely, transient periods of apnea and normal breathing leading to terminal apnea and asystole, with the severity of dysfunction potentially relating to sleep states.^3^ Hypoxia—abnormally low concentrations of oxygen in the blood—accompanies postconvulsive central apnea^4^ and hypoventilation,^5^ both of which can occur during and after generalized tonic‐clonic seizures (GTCS), and potentially contribute to the injury found later in SUDEP victims. Intermittent hypoxic exposure results in neuronal injury to central structures that are essential for recovery from profound hypotension or apnea (eg, Purkinje cells and the fastigial nuclei of the cerebellum in rats).^6^ Conditions that lead to sustained and intermittent hypoxia, such as congenital central hypoventilation syndrome, lead to damage to the posterior thalamus,^7^ an area that contributes to appropriate breathing in oxygen challenges,^8^, ^9^ as well as damage to the midbrain and cerebellar areas that mediate CO_2_ responses.^10^ Notably, both posterior thalamic gray matter volume loss and marked cerebellar injury appear in those who later have SUDEP and living patients at high risk.^1^

Several mechanisms of cell structure damage could accompany hypoxia. The processes include hyperexcitation accompanying neural activation during ictal processes, ultimately starving neurons if excitation is maintained without adequate perfusion.^11^, ^12^ Such a scenario would result in greater injury following repeated or prolongation of seizure exposure. If injury were to appear in cardiovascular or respiratory regulatory sites, the damage might be progressive from positive feedback processes.

The goal of the present study was to assess regional brain morphometric (gray and white matter volume) alterations relative to the extent of reductions in peripheral capillary oxygen (SpO_2_) saturation (hypoxia) as well as duration of hypoxia in patients with GTCS. Such an assessment would assist characterization of neuronal injury associated with hypoxia, enhance understanding of processes underlying brain structural alterations in SUDEP, and clarify strategies that might be incorporated to prevent neural injury during ictal events.

## 2 METHODS

### 2.1 Subjects

Subjects were identified from a database of patients recruited as part of an ongoing prospective investigation into autonomic and imaging biomarkers of SUDEP (the Center for SUDEP Research; CSR) conducted at University College London (UCL) and Case Western Reserve University (CWRU). The study was approved by the research ethics committee (19/SW/00071) and all subjects gave written informed consent. Included subjects were those with a GTCS captured during their noninvasive epilepsy monitoring unit (EMU) admission, peri‐ictal pulse oximetry data around the time of GTCS (a window of at least 1 minute prior to seizure onset, and 3 minutes post‐ictal), and a T1‐weighted MRI scan. The exclusion criteria were incomplete physiological data (missing or incomplete pulse oximetry data), previous neurosurgery, large brain lesions, incomplete clinical data, incomplete (or artifact‐ degraded) MRI scans. We additionally excluded those with a known long‐term history of phenytoin use, due to the known brain structural alterations associated with its use.^13^

Twenty‐two subjects (12 from UCL, 10 from CWR) were included in the study. A group of age‐ and sex‐comparable healthy controls, scanned at UCL, were included for comparisons with patient cohorts. Group characteristics were compared using nonparametric statistical tests in IBM SPSS v25 (Armonk, NY: IBM Corp). Clinical characteristics of groups can be found in Table 1. Two of the patients succumbed to SUDEP after enrollment. Although not included in the statistical analysis as a sub‐group (due to such a small sample size), details regarding these cases, and the potential relevance to the results observed here, are outlined in the discussion.

**TABLE 1:** Group demographics and clinical characteristics

Characteristics	HC (n = 43)	*GTCS* (n = 22)
Age, y (mean ± SD)	35.3 ± 12.9	36.9 ± 13.1
sex (M:F)	26:17	9:13
Disease duration, y (mean ± SD)		18.09 ± 11.1
GTCS/y (mean ± sd)		18.09 ± 24.9
Number AEds (mean ± sd)		2.86 ± 0.9
TBV, mL (mean ± SD)	1180.1 ± 118.4	1121.8 ± 105
Degree of SpO_2_ loss		
Median (%)		24
Range (%)		7‐64
Hypoxemia duration		
Median (s)		95
Range (s)		21‐415
Seizure‐onset zone (count)		
Focal > bilateral‐tc		18
Generalized onset		4

Abbreviations: AEDs, anti‐epileptic drugs; GTCS, generalized tonic‐clonic seizure; HC, healthy control; hypox, hypoxemia; mL, milliliter; SD, standard deviation; SpO_2_, peripheral oxygen saturation; TBV, total brain volume; TC, tonic‐clonic.

### 2.2 Identification and definition of hypoxia

Subjects underwent continuous SpO_2_ monitoring during a prolonged video‐EEG (electroencephalography) investigation, during which their habitual seizures were captured (details of seizure‐onset zones can be found in Table 2). SpO_2_ was measured using pulse oximetry (NONIN 8000J [Plymouth, MN, USA]). Measurements were obtained from the onset of the generalized phase until SpO_2_ recovered to baseline. Oxygen was administered in all subjects. Hypoxia duration was calculated as the length of time during which SpO_2_ was below 94%, in seconds (s). The *GTCS* group was further split into two groups for subanalyses: those in whom oxygen desaturations reached below 75% (*GTCS‐hypox‐severe*; n = 10), indicating severe hypoxia; and those in whom SpO_2_ remained above 75% (*GTCS‐hypox‐mild/moderate*; n = 12), indicating mild‐moderate hypoxia.^14^ Details of these sub‐groups can be found in Table 2.

**TABLE 2:** Demographics and clinical characteristics of the GTCS hypoxemia subgroups

Characteristics	Hypox mild/moderate (n = 11)	Hypox severe (n = 11)	*p*‐value
Age, y (mean ± SD)	38.1 ± 12.1	35.4 ± 14.7	0.50
sex (M:F)	5:6	4:7	0.47
disease duration (y, mean ± SD)	19.9 ± 11.2	15.9 ± 10.6	0.25
GTCS/y (mean ± sd)	23.8 ± 28.6	11.4 ± 19.2	0.42
Number of AEds (mean ± sd)	2.75 ± 1.1	3.0 ± 0.8	0.67
TBV, mL^3^ (mean ± SD)	1106 ± 109.6	1140.7 ± 101.6	0.28
hypox duration (s, mean ± sd)	169.5 ± 117.2	81.9 ± 38.4	0.31
Extent hypox (%, mean ± sd)	13.3 ± 6.1	41.9 ± 15	<0.001*
GTCS duration (s, mean ± sd)	44.7 ± 17.3	47.9 ± 18.0	0.68
Seizure onset/type (count)			
Focal > bilateral t‐c	9	9	NA
Generalized onset	2	2	NA
data from two SUDEP cases	Hypoxemia duration (s)	Hypoxemia extent (%)	
Case one	385	10	
Case two	95	55	

Abbreviations: AEDs, anti‐epileptic drugs; GTCS, generalized tonic‐clonic seizure; Hypox, hypoxemia; ml, milliliters; s, seconds; TBV, total brain volume; T‐C, tonic‐clonic.

In addition, we calculated the differences in SpO_2_ between baseline and the lowest value recorded, giving an individual measure of peri‐ictal oxygen desaturation (extent of SpO_2_ loss). We used this measure as a covariate of interest in region of interest (ROI) analyses to explore associations between regional brain volumes and oxygen desaturation. For subjects with more than one GTCS recorded, the lowest SpO_2_ measurements were averaged across each seizure before subtracting from averaged baseline, and durations of hypoxia (s) also averaged across seizures. Baseline SpO_2_ readings were obtained during periods of resting inter‐ictal wakefulness. Correlations were also performed between duration of hypoxia, extent of oxygen desaturation, and GTCS frequency. Duration and extent of hypoxia were also explored in relation to state at onset (ie, asleep vs awake).

### 2.3 MRI acquisition

High‐resolution three‐dimensional (3D) T1‐weighted images were acquired on a 3.0T GE MR750 at University College London (London, United Kingdom), and a 3.0T Siemens Skyra at Case Western Reserve University Hospitals (Cleveland, Ohio, United States). Image acquisition parameters (UCL/UH) were as follows: FOV (mm) = 224 × 256 × 256/230 × 173 × 230; acquisition matrix = 224 × 256 × 256/256 × 192 × 256; voxel size (mm) = 1.0 × 1.0 × 1.0/0.7 × 0.9 × 0.7; TR (repetition time, ms) = 7.4/7.3; TE (echo time, ms) = 3.1/2.38; TI (inversion time, ms) = 400/900; flip angle (degrees) = 11/9.

### 2.4 Image analysis

#### 2.4.1 Voxel‐based morphometry (VBM)

To explore brain‐wide differences in gray and white matter volume across groups, we employed voxel‐based morphometry (VBM) using the computational anatomy toolbox (CAT12; www.neuro.uni‐jena.de/cat/), in SPM12 (Statistical Parametric Mapping; http://www.fil.ion.ucl.ac.uk/spm) and MATLAB 2017b (MathWorks).

Images were denoised using the spatial‐adaptive nonlocal means filter and normalized to MNI152 template space, before being segmented into gray matter (GM), white matter (WM), and cerebrospinal fluid (CSF) classes. Modulated gray and white matter segmentations underwent thresholding (absolute, 0.2) to avoid partial‐volume effects, and z‐score normalization (−mean/standard deviation of the overall image intensity) to correct for potential intensity differences related to using data from multiple scanners. Z‐score maps were then smoothed with an 8 mm full‐width‐at‐half‐maximum (FWHM) Gaussian kernel before statistical analysis in SPM. For each subject, gray and white matter volumes were combined to estimate total brain volume, which was used as a covariate in subsequent statistical modelling. Two‐sample *t* tests were used for group comparisons in SPM, with age, sex, and total brain volume as covariates. Reported *P*‐values were family‐wise error rate (FWER)–corrected (largest cluster at *P* < .05).

#### 2.4.2 Region of interest analysis

Volumetric measurements were performed on 146 ROIs covering the entire brain to compare volumes across sub‐groups and quantify relationships between regional volumes and peri‐ictal oxygen desaturation and hypoxia duration. Each subject's (non‐normalized and bias‐corrected) T1 image was segmented and parcellated into 146 brain regions using GIF.^15^ Normalized volumes were obtained by multiplying the number of voxels in each ROI by the voxel volume, and dividing them by the total brain volume. ROI volumes were *Z*‐score normalized before comparison.

For structures where an appropriate ROI parcel was not available using GIF (eg, the periaqueductal gray [PAG]), volume of these structures was approximated in each subject. This was achieved by isolating the corresponding region‐specific cluster from the group SPM contrast map (ie, *GTCS‐hypox‐severe* < *HC*), which was then used to generate an explicit mask from which mean gray or white matter values were extracted from each subject's (gray or white) tissue probability map. These procedures were carried out in Matlab using Marsbar and SPM functions.

Regional volumes were compared across all sub‐groups using a multi‐variate analysis of covariance (MANCOVA) and post hoc permutation two‐sample *t* tests (with covariates age, sex, and total brain volume; 10 000 permutations). In addition, effect sizes, using Cohen's *D*, were calculated for significant group effects. Partial correlations between regional volumes and extent of oxygen desaturation/hypoxia duration were performed, accounting for GTCS frequency, disease duration, and total brain volume. Covariates (eg, GTCS frequency and disease duration) were also explored as variables of interest to highlight any associations with regional volumes. Because age and disease duration are correlated, these variables were entered in separate models to avoid problems with multicollinearity. ROI‐based statistical tests, including FWER *P*‐value corrections, were carried out using R (www.cran.r‐project.org).

## 3 RESULTS

### 3.1 Demographic, clinical, and hypoxia characteristics

Comparisons of *GTCS‐hypox‐severe* vs *GTCS‐hypox‐mild/moderate* group characteristics revealed no significant differences for age, sex, disease duration, GTCS per year, number of AEDs, and total brain volume (corrected by age)— see Table 1.

GTCS frequency correlated positively with duration of hypoxia (*r* = .6, *P* = .001), after controlling for disease duration. Seizure duration (generalized tonic‐clonic phase) was not significantly associated with hypoxia duration (*r* = −.1, *P* = .66) or extent (*r* = −.08, *P* = .70). Two cases of SUDEP were identified in the GTCS‐hypoxia group (see Table 2 for details).

In subjects with severe hypoxia, extent of SpO_2_ desaturation was, on average, 7% greater (*P* > .05) in those whose seizures arose from sleep. Hypoxia durations were 9.6 seconds longer (*P* > .05) in those with sleep‐bound seizures, when considering only subjects with severe hypoxia; durations were, on average, 31 seconds shorter (*P* > .05) in those with seizures arising from wakefulness among subjects with mild/moderate peri‐ictal hypoxia.

### 3.2 Data from two SUDEP cases

Two confirmed SUDEP cases were identified in this cohort. Both exhibited peri‐ictal hypoxia. The first case had a 10% extent of hypoxia (above 75%; mild‐moderate), but with a longer duration (385 seconds; the longest observed). This individual also had post‐convulsive central apnea. The second case showed a shorter hypoxemic duration (95 seconds), but more profound desaturation (55%; the third highest observed).

### 3.3 Voxel‐based morphometry

#### 3.3.1 Gray matter

**FIGURE 1:**
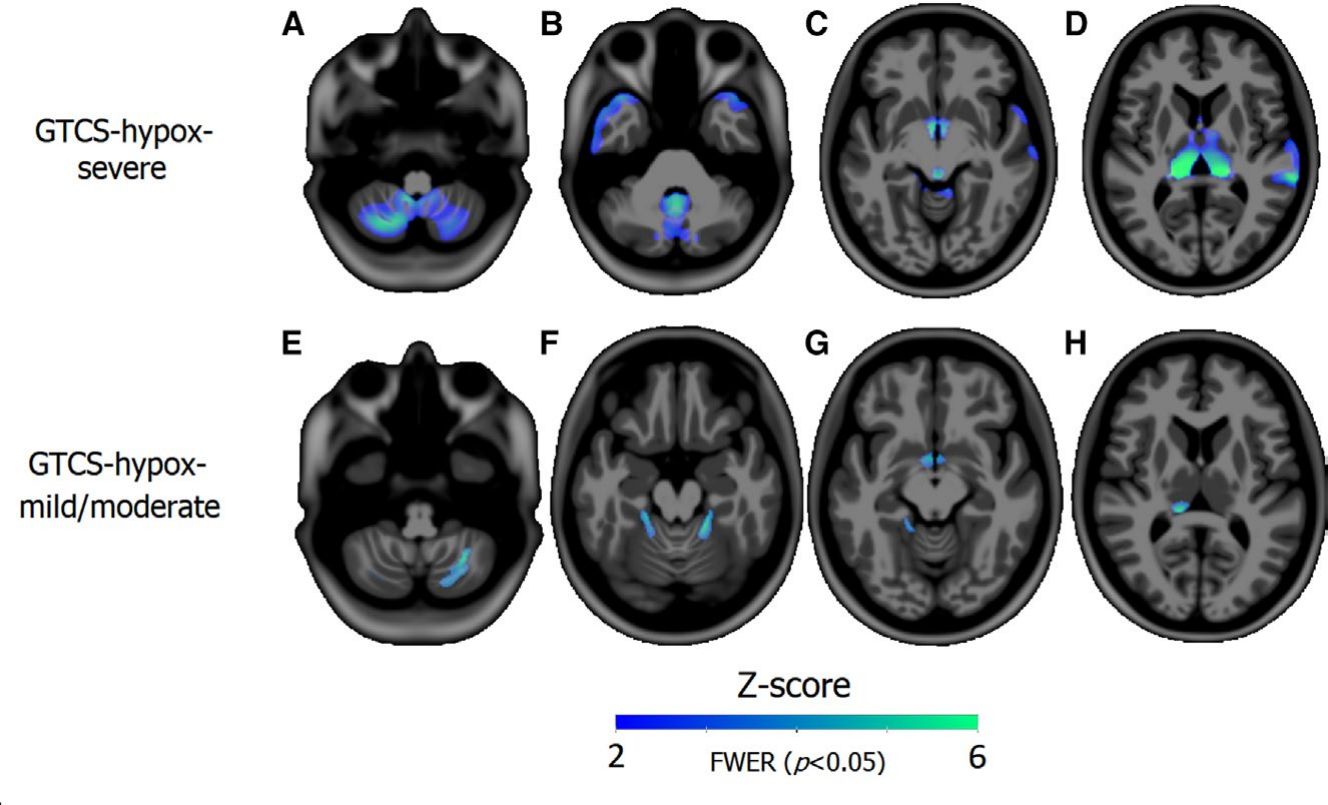
Reduced gray matter in generalized tonic‐clonic seizures (GTCS)‐hypox‐severe (top) and GTCS‐hypox‐mild/moderate (bottom) compared with healthy controls (HC). Gray matter alterations in GTCS‐hypox‐severe compared with HC: volume loss appears in the cerebellum (A), vermis and temporal pole (B), hypothalamus and periaqueductal gray (PAG) (C), and bilateral thalamus (D). Gray matter volume loss of bilateral lateral cerebellum, hypothalamus, and left posterior thalamus in GTCS‐hypox‐mild/moderate compared with HC (E‐H). GTCS, generalized tonic‐clonic seizures

Compared with healthy controls, *GTCS‐hypox‐severe* showed reduced GM volume in the bilateral cerebellum, vermis, temporal pole, PAG, hypothalamus, thalamus, and right superior temporal gyrus (Figure 1A‐D). *GTCS‐hypox‐mild/moderate* showed GM loss in the lateral cerebellum, bilateral hypothalamus, and left posterior thalamus when compared with healthy controls (Figure 1E‐H).

#### 3.3.2 White matter

**FIGURE 2:**
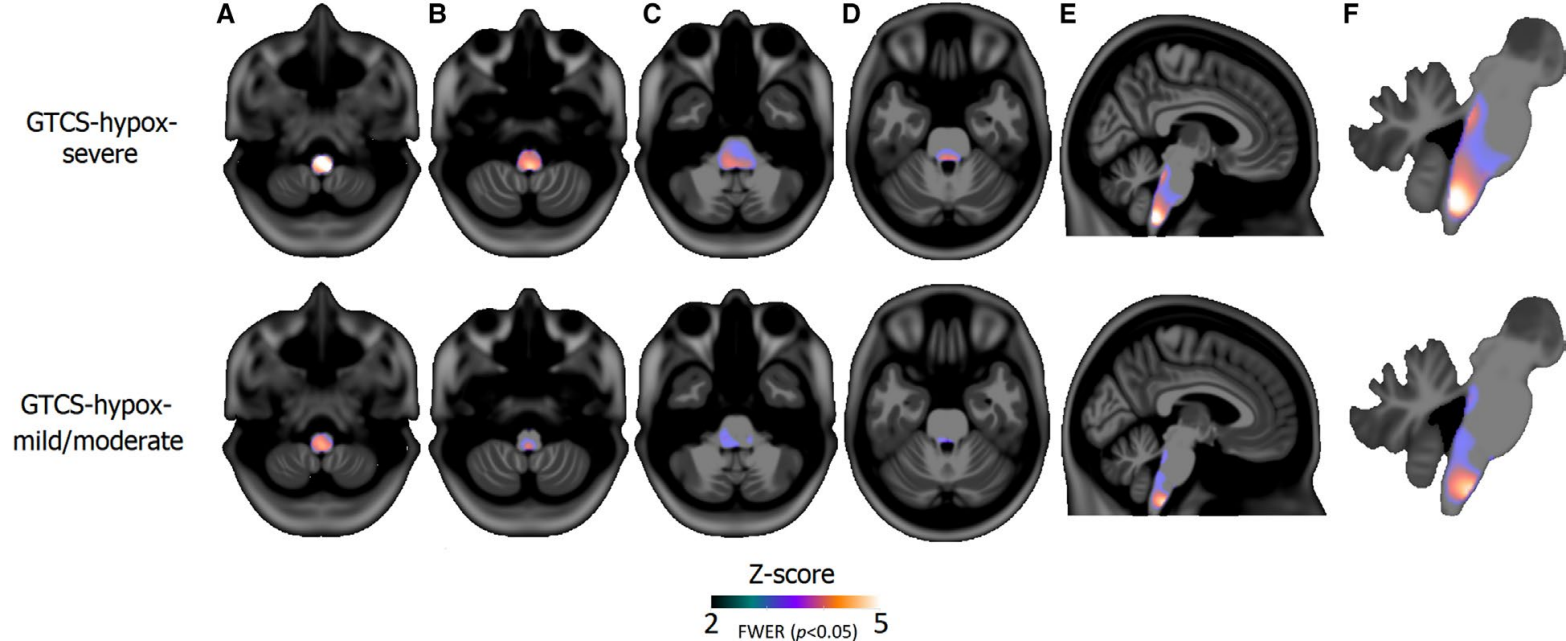
Reduced white matter volume in GTCS‐hypox‐severe (top row) and GTCS‐hypox‐mild/moderate (bottom row) compared with healthy controls (HC). Significant white matter volume loss appeared in the medulla of the lower brainstem (A,B and E,F) and parabrachial area of the pons (C‐F) in both groups, although to a lesser extent in GTCS‐hypox‐mild/moderate. GTCS, generalized tonic‐clonic seizures

*GTCS‐hypox‐severe* showed reduced WM volume in the medulla (including dorsal and ventral respiratory groups; Figure 2A,B,E,F) and the dorsal pons (including the parabrachial complex; Figure 2C‐F). *GTCS‐hypox‐mild/moderate* showed loss in similar sites, although to a lesser extent, particularly around the dorsal pons (Figure 2, bottom row).

### 3.4 ROI analysis

#### 3.4.1 Sub‐group differences

**FIGURE 3:**
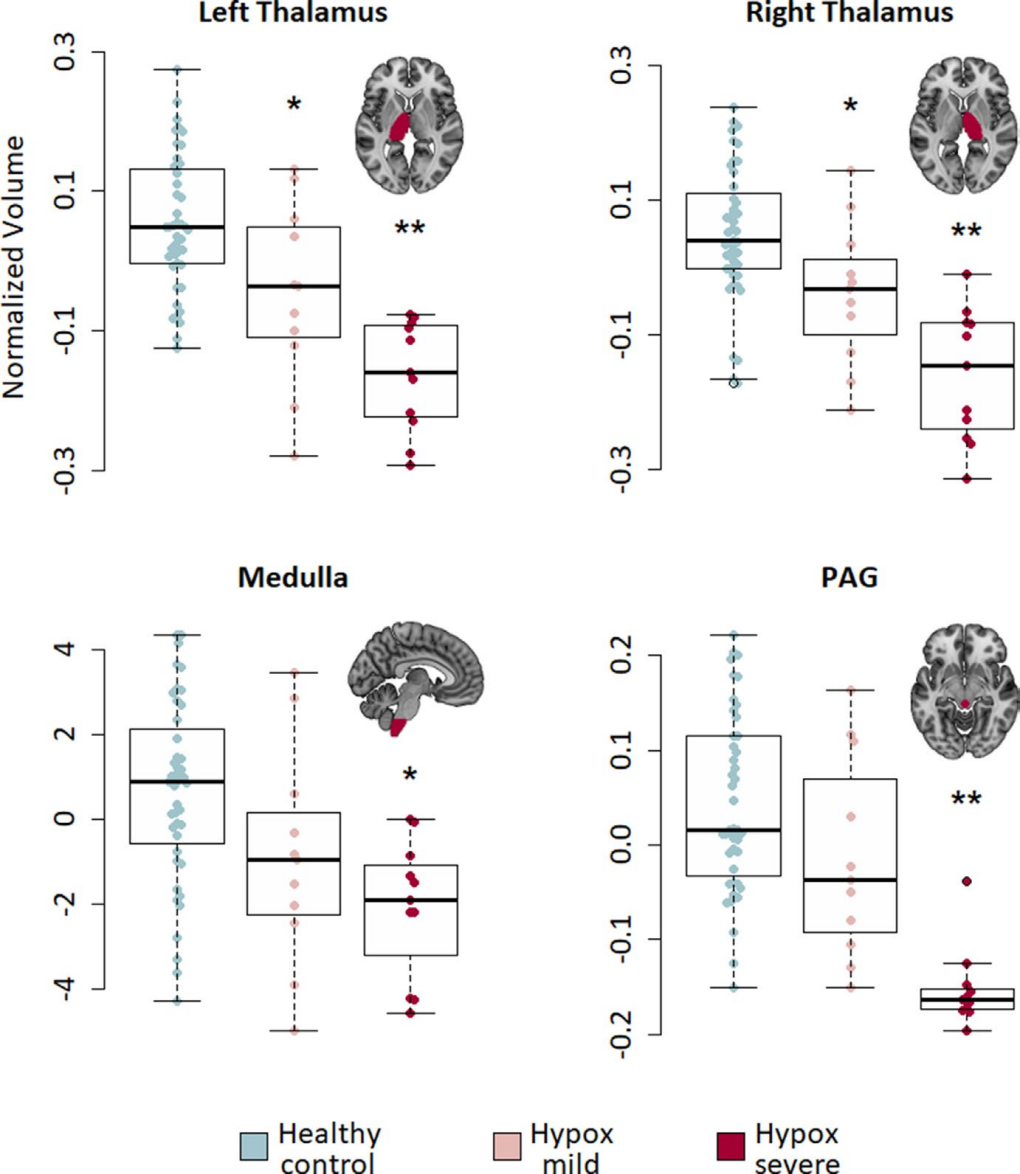
Region of interest (ROI) volumes across groups. Swarm plots with boxplot overlay showing the distributions of PAG (top left), medulla (top right), and thalamic (bottom left [L] and bottom right [R]) volume across groups. PAG, periaqueductal gray. *P*‐values are FWER‐corrected. *Significant compared to healthy controls only (*P* < .05 FWER). **Significant compared to both healthy controls and GTCS‐hypox‐mild/moderate

ROI volumes of structures showing that significant group‐level VBM effects were compared across the sub‐groups. Main effects were found for left (*F* = 15.5, *P* = 5.12E‐08) and right (*F* = 14.5, *P* = 1.34E‐07) thalamus, PAG (*F* = 8.1, *P* = <.0001), and medulla (*F* = 6.5, *P* = .002). All ROI volume results are displayed in Figure 3.

Left and right thalamic volumes were reduced in both sub‐groups compared with healthy controls (*GTCS‐hypox‐mild/mod* [left: *t* = 3.6, *P* = .001, *D* = 0.96/right: *t* = 2.9, *P* = .005, *D* = 0.89]; *GTCS‐hypox severe* [left: *t* = 6.4, *P* = 4.43E‐08, *D* = 2.3/right: *t* = 6.2, *P* = 9.66E‐08, *D* = 2.1]).

Thalamic volumes in *GTCS‐hypox‐severe* were also reduced when compared with *GTCS‐hypox‐mild/mod* (left: *t* = 2.6, *P* = .016, *D* = 1.1/right: 3.4, *P* = .003, *D* = 1.2).

PAG volume was reduced in *GTCS‐hypox‐severe* when compared with healthy controls (*t* = 5.6, *P* = 7.58E‐07, *D* = 2.2) and *GTCS‐hypox‐mild/mod* (*t* = 2.8, *P* = .004, *D* = 2.1). GTCS‐hypox‐mild/mod had reduced PAG volume compared with healthy controls only, but this difference did not reach statistical significance (*t* = 1.9, *P* = .07, *D* = 0.7).

Medullary volume was reduced in *GTCS‐hypox‐severe* (*t* = 4.0, *P* = <.001, *D* = 1.2) compared with healthy controls.

#### 3.4.2 Partial correlations

**FIGURE 4:**
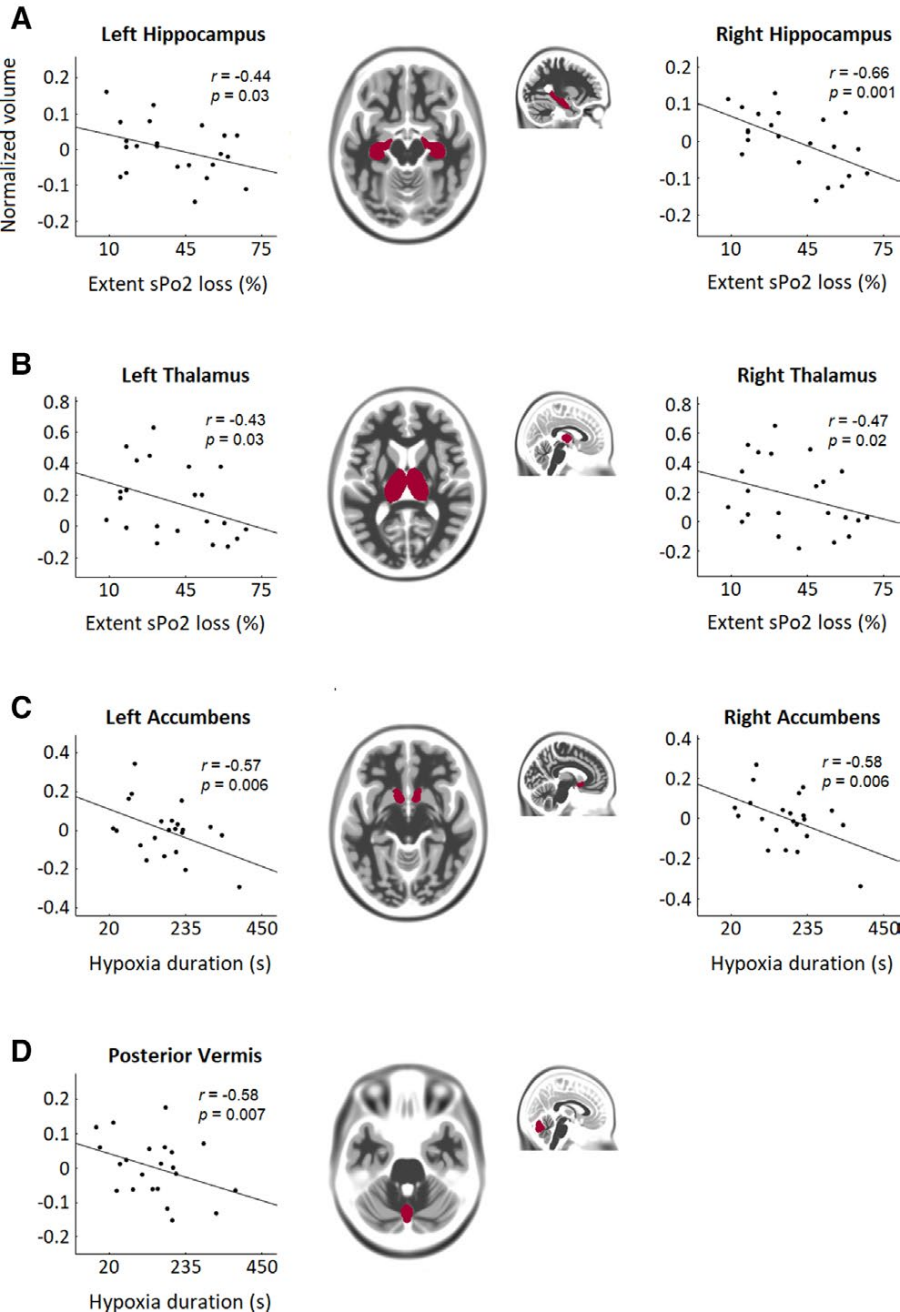
Partial correlations between regional volumes and extent of SpO_2_ loss/hypoxia duration. Regions for which significant correlations with extent of SpO_2_ loss/hypoxia duration were found are overlaid in magenta on a template brain in MNI space. Hippocampal (A) and thalamic (B) volumes were negatively associated with extent of SpO_2_ loss. Accubmens nuclei (C) and posterior vermis (D) volumes were negatively associated with hypoxia duration. *r*, Partial correlation coefficient. Covariates were disease duration and total brain volume. Volume scale represents normalized *z*‐score values. *P*‐values were FWER‐corrected

Negative partial correlations emerged between extent of SpO_2_ desaturation and left (*r* = −.429, *P* = .03) and right (*r* = −.468, *P* = .02) thalamus, and left (*r* = −.435, *P* = .03) and right (*r* = −.66, *P* = .001) hippocampus (Figure 4A,B). Negative partial correlations appeared between duration of hypoxia and the posterior vermis (*r* = −.58, *P* = .007) and left (*r* = −.57, *P* = .006) and right (*r* = −.58, *P* = .006) accumbens (Figure 4C,D). GTCS frequency correlated positively with left (*r* = .67, *P* = .003) and right (*r* = .43, *P* = .04) thalamic GM volume, only when considering all GTCS subjects in one group.

## 4 DISCUSSION

### 4.1 Overview

Our findings show strong associations between gray and white matter volume abnormalities and extent (and duration) of hypoxia in patients with GTCS.

Widespread volume loss appeared in subjects whose seizures were accompanied by severe hypoxia, especially in GM sites previously shown to exhibit loss in patients with SUDEP and those at highest risk (thalamus, cerebellum, vermis, and PAG)^16^. In addition, we found GM loss in the hypothalamus and temporal pole, and white matter reductions in the medulla and pons of the brainstem, regions known to be associated with SUDEP.^17^ Subjects with mild/moderate hypoxia showed more conservative reductions in GM, in the bilateral hypothalamus, lateral cerebellum, and left posterior thalamus.

Correlational analyses indicated an association between hypoxia and volumes of specific sites that serve significant blood pressure and breathing roles: the posterior vermis,^18^ thalamus, ^7^, ^8^, ^9^ and hippocampus.^19^, ^20^ These sites exhibit volume loss in previous imaging studies of SUDEP and at‐risk populations,^1^, ^2^ as well as in other cohorts involving respiratory dysfunction.^7^ Data from two SUDEP cases indicate a potential role of hypoxia extent and duration in SUDEP, although further work involving larger samples is needed.

### 4.2 Volume loss associated with hypoxia

Volume loss of the thalamus, PAG, and medulla followed a progressive pattern, in association with increasingly elevated peri‐GTCS hypoxia across groups; subjects with severe hypoxia showed the most extensive loss within these sites, whereas subjects with mild/moderate hypoxia showed less. Thalamic and brainstem volume loss is well described in SUDEP and those at risk, including GTCS patients.^1^ We found here that hypothalamic GM is also reduced in GTCS with hypoxia, an outcome not reported previously in those at risk for SUDEP.

The thalamus, particularly its posterior aspect, plays roles in mediating oxygen sensitivity^7^, ^8^, ^21^ as well as CO_2_,^10^ and directs and receives respiratory information to and from the medulla,^22^ where the dorsal and ventral respiratory groups for inspiratory and expiratory timing are located.^23^ The parabrachial pons, which showed loss in GTCS‐hypox‐severe and GTCS‐hypox‐mild/moderate (although to a lesser extent) compared with healthy controls, is critical for respiratory phase switching, and receives projections from the central amygdala, an important area for respiratory drive. The parabrachial pons provides synaptic medullary input for shaping and adaptation of breathing patterns,^24^ and is involved in hypoxic depression of breathing in fetal rabbits.^25^ The PAG assists recovery from cardiorespiratory dysfunction, deficient responses of which can result in SUDEP.^26^ Portions of the hypothalamus play roles in a variety of regulatory processes, including thermoregulation (and thus, breathing, considering the role of thermal drive on respiration^27^), sympathetic outflow, and thus, blood pressure regulation^28^ and arousal.^29^

Because volume changes of certain structures followed a progressive, seemingly linear (in the direction of elevated per‐GTCS hypoxia), pattern of loss across groups with hypoxia, a logical next step was to determine whether the magnitude of site‐specific volume reductions could be explained by the extent or duration of hypoxia, as assessed with correlation analyses. The extent of hypoxia correlated negatively with bilateral thalamic and hippocampal volumes, indicating that reduced volumes of these structures are accompanied by more‐pronounced hypoxia. Although a causal link cannot be established from these findings (since changes are associative and correlational), volume loss may reflect gliosis stemming from repeated hypoxic insults arising from frequent convulsions.^30^ Hypoxia certainly has the potential to damage these structures; failure of oxygenation or reduced perfusion resulting from hypoxia‐induced vascular constriction to cells undergoing high metabolic demands during ictal events could establish a scenario for excitotoxic injury.^31^, ^32^ However, regardless of injury mechanisms, volume loss to the thalamus, medulla, and pons may represent functional impairment to sites and networks involved in central respiratory modulation and cardiorespiratory recovery in those with severe hypoxia.

Assessments of hypoxia duration revealed negative correlations with volume of the accumbens (bilaterally) and posterior vermis, suggesting that longer hypoxia durations are linked to greater volume loss in these areas. Intermittent hypoxia in early life damages the posterior vermis, including the fastigial nuclei^6^; similar processes may contribute to volume loss here, whereby prolonged hypoxia episodes lead to cerebellar GM injury.

The nucleus accumbens forms part of the ventral striatum and plays roles in learning and reward circuitry, with strong dopaminergic and serotonergic inputs.^33^ A role in depression is also known; deep brain stimulation of the accumbens reduced anxiety and depression ratings in treatment‐resistant depression.^34^ We note that the accumbens is not known to directly participate in central autonomic or respiratory regulation, although reduced volume associated with extended durations of hypoxia might indicate damage via sustained hypoxia.

### 4.3 Clinical variables

GTCS frequency was positively associated with hypoxia duration. If successive GTCS leads to more prolonged hypoxic exposure, repeated GTCS may indirectly contribute to volume alterations in individuals with hypoxia, if the mechanisms of injury can be confirmed as arising directly from low oxygen or indirectly through excitotoxic processes. Such a scenario could lead to the observed volume loss in such areas as the vermis and hippocampus—regions that are highly susceptible to excitotoxic cell loss,^7^, ^35^ which showed reductions in volume in association with greater hypoxia duration in this study.

Bilateral thalamic volumes correlated positively with GTCS frequency in GTCS patients with hypoxia (when combining GTCS‐hypox‐severe and GTCS‐hypox‐mild/moderate groups). These data provide evidence for the possibility that thalamic volume loss in GTCS patients, and associated with hypoxia, cannot be attributed solely to GTCS frequency. Notably, no other clinical variables showed meaningful or significant correlations with regional structure volumes, including disease duration, which was associated earlier with thalamic volume loss.^2^

We also explored hypoxia duration and extent in relation to state at seizure onset (asleep or awake) in light of the known alterations to autonomic and respiratory processes during sleep, and the association between sleep and SUDEP. In subjects with severe hypoxia, extent of SpO_2_ desaturation was, on average, 7% greater in those whose seizures arose from sleep, although this difference was not statistically significant. Hypoxia durations were 9.6 seconds longer in those with sleep‐bound seizures, when considering only subjects with severe hypoxia; durations were, on average, 31 seconds shorter in those with seizures arising from wakefulness among subjects with mild/moderate peri‐ictal hypoxia. Although none of the above findings were statistically significant, they highlight important interactions between state at seizure onset and hypoxia, which merit further study in future investigations with larger samples.

### 4.4 Relevance to SUDEP research: hypoxia data from two cases

Two confirmed SUDEP cases were identified in this cohort. Both exhibited peri‐ictal hypoxia. The first case had a 10% extent of hypoxia (above 75%; mild‐moderate), but with a longer duration (385 seconds; the longest observed). This individual also had post‐convulsive central apnea. The second case showed a shorter hypoxemic duration (95 seconds), but more profound desaturation (55%; the third highest observed). Although few inferences can be derived from this small number of observations, highlighting the characteristics of these cases is worthwhile given the rarity of SUDEP and the objectives of this study. Of note, many of the observations of peri‐ictal hypoxia were based on one seizure alone—durations and extents of hypoxia may vary on a within‐subject basis, and are influenced by, or interact with, other factors (such as state at seizure onset, time of day, or other peri‐ictal autonomic/ respiratory manifestations), all of which require further investigation. Both duration and extent of hypoxia may be relevant for SUDEP, and may involve separate mechanisms (eg, apnea vs hypoventilation).

### 4.5 Considerations and limitations

#### 4.5.1 Interpretation of volume loss and links to SUDEP

As with most observational studies, the level of inference available from our findings is associative only. Therefore, it cannot be claimed from the current study that hypoxia causes the observed volume alterations. In addition, although hypoxemic mechanisms may be a key risk factor underlying SUDEP, this possibility remains speculative, and we can only suggest the possibility that volume alterations related to hypoxia may be linked with processes involved in SUDEP. However, the findings outline important relationships and provide insights into potential mechanisms of volume loss, and indicate target sites for future studies and potential interventions.

#### 4.5.2 Measurements and mechanisms of hypoxia

SpO_2_ measurements were assessed via pulse oximetry to establish hypoxia values. Although such measurements correlate sufficiently well with “gold‐standard” arterial oxygen saturation (SaO_2_) monitoring to enable useful clinical determinations, SpO_2_ is inferior to SaO_2_ in terms of artifact susceptibility and measurement accuracy. More‐focused studies addressing hypotheses involving hypoxia would benefit from superior approaches to measuring oxygen saturation.

Oxygen was administered to subjects during or after seizures while in the epilepsy‐monitoring unit; however, such an intervention is rarely, if ever, performed at home or in other settings in which an individual might experience a seizure. This raises the concern that hypoxia, despite additional oxygen, in these individuals might indicate an increased propensity for reductions during or after seizures, which may be even more severe at home where oxygen cannot be administered. In addition, if respiratory effort is severely reduced or diminished, the effect of additional oxygen may be reduced or ineffective.

The current study did not assess the pathways underlying hypoxia during GTCS, which may be key to ascertaining the pathophysiological processes leading to SUDEP. Hypoxia can arise from central^4^ or obstructive apnea,^36^, ^37^ or from sustained hypoventilation reflecting reduced drive to breathe.

The interpretations here are based solely on observations of reduced oxygen saturation, and not the processes by which those desaturations emerged. Additional studies might determine the precise mechanisms that lead to hypoxia around the time of seizures, particularly during and after GTCS. As is a future objective of the current work, investigation of additional peri‐ictal autonomic and breathing measurements, such as heart rate and respiratory rate and depth, would be key for future studies to assess the origins of hypoxia and clarify the nature of injury. Such insights would significantly enhance our understanding of cardiorespiratory dysfunction, and related ictal neural damage, such as vascular microspasm leading to reduced tissue perfusion,^38^ in epilepsy and the events leading to SUDEP.

#### 4.5.3 Cohort

Subjects were selected based on the availability of recorded breathing parameters and imaging, and thus were not confined to a specific sub‐group of epilepsy, such as those with epilepsy of temporal lobe origin. As such, the cohort is represented by an inhomogeneous group of patients and the resulting data are confounded by variance related to differing disease etiologies, epilepsy types, seizure‐onset zones, and imaging findings. Ultimately, the findings do not provide insight into associations between hypoxia and other aspects such as epileptogenic zones, owed to a limited sample size.

In addition, only patients with GTCS were included in the current study, and the absence of focal seizure sub‐grouping means interpretation is limited to these patients. A major future objective of the present study is to examine hypoxia‐related structural alterations in subjects with focal seizures as well. This would be particularly critical to determine the nature of damage to such structures as the hippocampus and thalamus, which are often both damaged in patients with focal seizures and could potentially reflect excitotoxic injury.

However, given the context that SUDEP is closely associated with GTCS incidence, relating findings to focal seizures remained a lesser priority here. Future studies might attempt to evaluate hypoxia and associated brain changes in focal seizures as well.

#### 4.5.4 Data from different scanners

Because the current study forms part of a larger multi‐center effort, data were derived from two sites—UCL and Case Western Reserve University—and, thus, two separate scanners. A major advantage of multi‐center studies is increased subjects numbers, and hence statistical power, particularly in the context of rare diseases and observations.

VBM measurements from longitudinal studies differ across multiple scanners, and even scanner upgrades.^39^ It is notable that in this study, as with others,^40^ scanner differences did not appear within sites showing significant effects of interest between disease groups. In addition, group membership of subjects from either site was approximately even, ensuring a balance of representatives across groups.

## 5 CONCLUSIONS

The extent of hypoxia during seizures in GTCS patients is related to the magnitude of brain morphological and volumetric alterations in sites that serve oxygen sensing or integration, breathing regulation, and cardiovascular recovery.

Our data are therefore consistent with a link between hypoxia and structural abnormalities in brain regions, which serve cardiovascular and breathing functions. Injury to these sites may reflect ongoing damage related to autonomic and respiratory disturbances in the context of seizures, which could modify central cardiovascular and respiratory processes when recovery from extreme breathing or cardiovascular challenges induced by GTCS is essential. The findings provide insights into processes underlying frequently described tissue injury in GTCS and narrow the focus for protective interventions.

## CONFLICT OF INTEREST

None of the authors have any conflict of interest to disclose. We confirm that we have read the Journal's position on issues involved in ethical publication and affirm that this report is consistent with those guidelines.
